# Supplementary material for: Oral Mucositis in Adult Cancer Patients Undergoing Chemotherapy: Six-Month On-Treatment Follow-Up
Source: J Clin Med. 2024 Sep 25;13(19):5723. doi: 10.3390/jcm13195723 (PMC11477337; doi:10.3390/jcm13195723)
Supplement: Supplementary file 1 [file jcm-13-05723-s001.zip › jcm-3184868-supplementary.pdf]

Supplemental material to:

Oral Mucositis in Adult Cancer Patients Undergoing Chemo-therapy: Six-Month On-Treatment Follow-Up

R code for Cox regression analysis

```
library("survival")
library("rms")
############### Cox regression ##############################
data6month <- read.csv("dataFile.csv")
data6month$TreatmentClass <- as.factor(data6month$TreatmentClass)
mod2.ulceration <- coxph(formula=Surv(Month,Lesions23) ~ protocolBin + age + sexM +
        I1initial + treatmentClass, data=data6month)
mod2.ulceration
summary(mod2.ulceration)
############### collinearity testing ##########################
cvif <- vif(mod2.ulceration)
cvif
```

Results

```
> summary(mod2.ulceration)
Call:

coxph(formula = Surv(Month, Lesions23) ~ protocolBin + age +
    sexM + I1initial + TreatmentClass, data = data6month)

  n= 55, number of events= 25

                     coef  exp(coef)   se(coef)      z Pr(>|z|)
protocolBin     -1.874e+00  1.535e-01  5.843e-01 -3.207  0.00134 **
age             -3.194e-02  9.686e-01  2.292e-02 -1.393  0.16348
sexM            -5.006e-01  6.062e-01  6.657e-01 -0.752  0.45207
I1initial       -3.810e-01  6.832e-01  3.148e-01 -1.210  0.22617
TreatmentClass2 -4.393e-01  6.445e-01  8.811e-01 -0.499  0.61807
TreatmentClass3  2.226e-01  1.249e+00  6.215e-01  0.358  0.72027
TreatmentClass4  2.793e-01  1.322e+00  6.491e-01  0.430  0.66699
TreatmentClass5  3.127e-01  1.367e+00  1.298e+00  0.241  0.80960
TreatmentClass6  1.581e+00  4.859e+00  1.190e+00  1.328  0.18414
TreatmentClass7 -1.532e+01  2.218e-07  4.589e+03 -0.003  0.99734
---
Signif. codes:  0 ‘***’ 0.001 ‘**’ 0.01 ‘*’ 0.05 ‘.’ 0.1 ‘ ’ 1

                exp(coef) exp(-coef) lower .95 upper .95
protocolBin     1.535e-01  6.514e+00   0.04884    0.4825
age             9.686e-01  1.032e+00   0.92601    1.0131
sexM            6.062e-01  1.650e+00   0.16442    2.2348
I1initial       6.832e-01  1.464e+00   0.36862    1.2662
TreatmentClass2 6.445e-01  1.552e+00   0.11461    3.6242
TreatmentClass3 1.249e+00  8.005e-01   0.36953    4.2233
TreatmentClass4 1.322e+00  7.563e-01   0.37050    4.7184
TreatmentClass5 1.367e+00  7.315e-01   0.10743   17.3964
TreatmentClass6 4.859e+00  2.058e-01   0.47139   50.0868
TreatmentClass7 2.218e-07  4.508e+06   0.00000       Inf

Concordance= 0.708  (se = 0.054 )
Likelihood ratio test= 13.82  on 10 df,   p=0.2
Wald test            = 12.94  on 10 df,   p=0.2
Score (logrank) test = 15.04  on 10 df,   p=0.1


> cvif
    protocolBin             age            sexM       I1initial TreatmentClass2
       2.087631        1.890322        2.230875        1.620932        1.419841
TreatmentClass3 TreatmentClass4 TreatmentClass5 TreatmentClass6 TreatmentClass7
       2.096189        2.286396        1.616210        1.322350        1.000000
```
